# Surgical outcomes of localization using indocyanine green fluorescence in breast conserving surgery: a prospective study

**DOI:** 10.1038/s41598-021-89423-w

**Published:** 2021-05-11

**Authors:** Eun-Gyeong Lee, Seok-Ki Kim, Jai Hong Han, Dong-Eun Lee, So-Youn Jung, Seeyoun Lee

**Affiliations:** 1 Department of Surgery, Center for Breast Cancer, Research Institute and Hospital, National Cancer Center, 323 Ilsan ro, Ilsandong-gu, Goyang-si, Gyeonggi-do 10408 South Korea; 2 Department of Nuclear Medicine, National Cancer Center, Goyang, South Korea; 3 Biostatistics Collaboration Team, Research Core Center, Research Institute of National Cancer Center, Goyang, South Korea

**Keywords:** Medical research, Oncology, Breast cancer

## Abstract

We investigated localization and safe resection margins for breast cancer patients undergoing breast conserving surgery (BCS) using ultrasound-guided indocyanine green fluorescence (ICG-F) marking. From April 2016 to March 2019, we prospectively enrolled 114 patients who underwent BCS using US-guided ICG-F marking and we compared these results with 300 patients who underwent BCS using US-guided skin marking from January 2012 to December 2016. Clinical features, identification rates, status of resection margins, and re-operation rates were analyzed. The ICG-F identification rate was 100% (114/114). The mean approach time for resection of the lesion ICG-F using group was about 13 min. The positive rate of frozen resection margins was 10.5% using ICG-F and 25.0% using sono-guided skin marking (*p* < 0.01). The rate of additional intraoperative resection was significantly lower in the ICG-F marking group compared to that in the sono-guided skin marking group (8.8% vs. 23.3%, *p* < 0.01). The rate of final positive resection margins was 3.5% in the ICG-F using group and 14.7% in the sono-guided skin marking group (*p* < 0.01). The rate of re-operation was 4.4% in the ICG-F using group and 4% in the sono-guided group (*p* = 0.79). At follow-up after the operation using ICG-F, no complications occurred. Using ICG-F during BCS could be a safe, sophisticated method for localization.

## Introduction

Mammography, one of the main screening methods for breast cancer, has become popular as the incidence of breast cancer continues to increase^1^. Breast conserving surgery (BCS) is the preferred surgical method for early breast cancer patients with single lesion. With an increasing number of small-sized lesions that are not palpable, there is an increasing demand for a localization method for surgery. Localization techniques for non-palpable breast lesions include conventional methods such as sono-guided skin markings and needle localization before surgery. Previous studies for non-palpable breast cancer showed that identification rates of tumors with sono-guided localization was 100% and rates of negative margins with sono-guided BCS was 89–96.3%^2,3^. In addition, there are various methods for localization such as charcoal localization, radioactive seed localization, and MRI-guided needle localization^4–6^. These localization techniques still have many limitations including patient discomfort, migration, radiation exposure, inability to reposition the seed, depth limitation, and cost^7^.

Recently, the use of indocyanine green fluorescence (ICG-F) for sentinel lymph node biopsy in breast cancer evaluations is gradually increasing, replacing the existing localization technique. The use of various localization methods is gradually increasing for various cancer surgeries, and replacing the existing localization techniques. There have been some reports of excisions which injected ICG-F into the intra-tumor for non-palpable lesions of breast cancer. Localized excisions using ICG-F have been shown to be possible as an alternative to previous existing methods^8–10^. However, a study of excisions using sono-guided peritumor ICG-F injections for breast cancer has yet to be published.

This study aimed to identify localization and determine whether it is possible to more easily and accurately remove breast cancer using a near-infrared fluorescent camera after injecting ICG-F surrounding a tumor.

## Materials and methods

### Study design

We planned a phase 2, interventional, prospective, case–control, single-center study. We included the patients with early breast cancer who were 18 years or older with stage Tis-T2, N0 and M0 primary breast cancer according to the American Joint Committee on Cancer (AJCC) staging system and patients who were candidates for BCS with an Eastern Cooperative Oncology Group (ECOG) performance score of 0 or 1. We excluded patients with inflammatory breast cancer and those who needed a total mastectomy, those who were pregnant or breast feeding women, people with a history of severe allergies to ICG, with iodine hypersensitivity, breast cancer of T3 or greater according to imaging findings, and the patients who had neoadjuvant chemotherapy.

From April 2016 to March 2019, we enrolled 114 patients who underwent BCS using US-guided ICG-F marking prospectively. We compared these results with a control group of 300 patients who underwent BCS using US-guided skin marking by the same surgeons from January 2012 to December 2016.

### Methods for injecting the materials and identification

**Figure 1:**
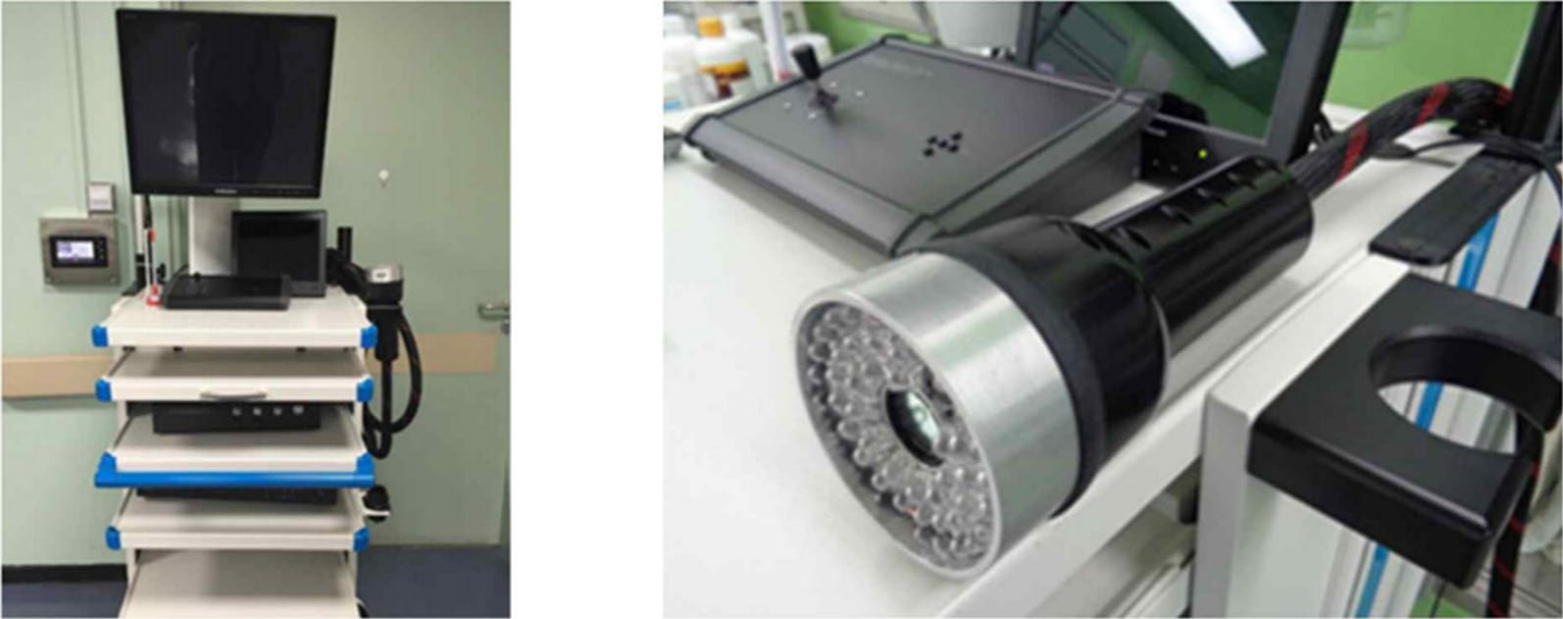
Near-infrared fluorescence imaging system (with a visual navigator and portable handy instrument).

**Figure 2:**
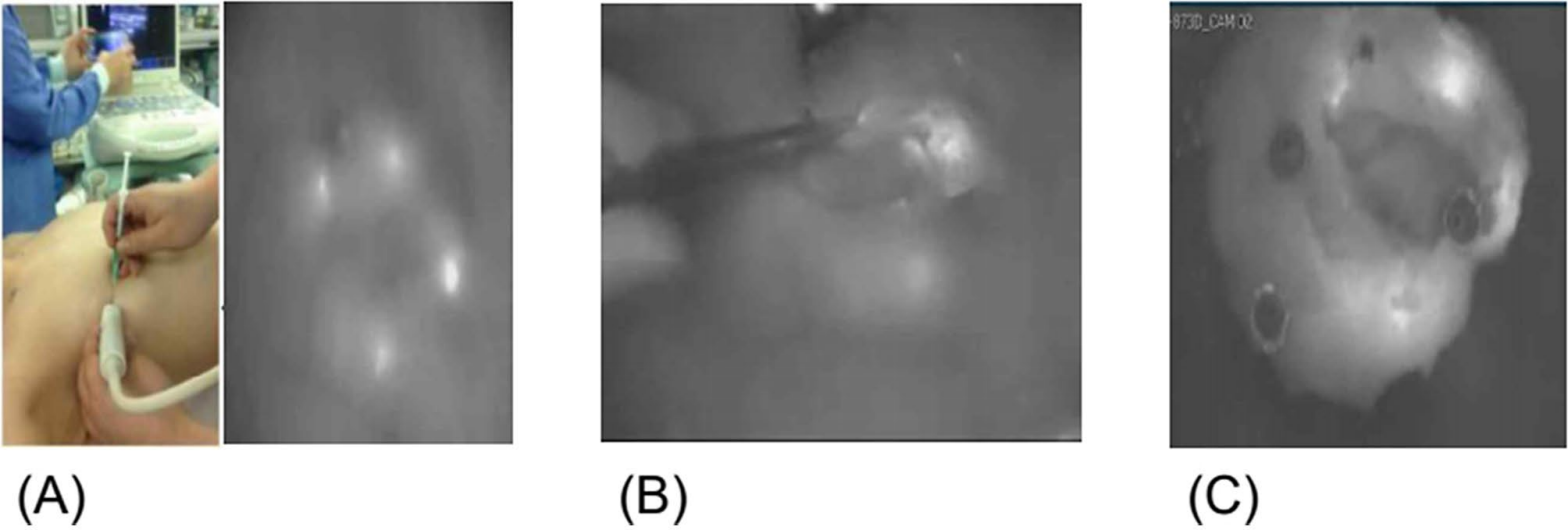
Localization by ICG injection (ICG injection into the target lesion under the guidance of ultrasonography). (**A**) The fluorescent agent is injected into 4–8 areas of the breast cancer resection surface using ultrasound before surgery. (**B**) During surgery, the surgeon finds and removes the fluorescent lesion using a near-infrared fluorescence camera. (**C**) After lesion removal, the completeness of the resection through a near-infrared fluorescent camera was confirmed.

Before the operation begins, we check the lesion area with ultrasonography, dilute 25 mg of ICG dye (Diano-green; Daichi Pharmaceutical, Tokyo, Japan) in 10 ml of distilled water and then inject 0.2 ml of this solution into 4–8 places of the malignant tumor resection surface under ultrasonography. Before the incision, we check and mark the location of the fluorescence-injected lesion on the skin through the near-infrared fluorescence camera. Next, we perform an incision at the location of the confirmed lesion, and during surgery, we find and remove the lesion that is highlighted through the near-infrared fluorescent camera. For the ICG-F display system, we used the previously used fluorescence imaging system (Visual Navigator; SH System, Gwangju, Korea) consisting of a small charge-coupled device camera with an integrated near-infrared (NIR) light source (energy, 2.4 W; wavelength, 740 nm). A band-path filter (820 nm) was used as the emission filter to collect NIR radiation and reject visible and excitation light (Fig. 1)^11^. After the lesion is removed, we checked if there was any remaining fluorescence displayed through a near-infrared fluorescent camera in the surgical field. If the fluorescence remains, the relevant part would be further resected during the surgery. During surgery, the removed specimen was checked for the completeness of the resection through a near-infrared fluorescent camera, and the pathological negative margin was confirmed by the results of the frozen diagnosis (Fig. 2).

### Margin status

All patients underwent intraoperative margin assessment by frozen section. If the result of resection during surgery indicated precancerous lesions such as atypical ductal hyperplasia, atypical lobular hyperplasia, and lobular cancer in situ, or confirmed as DCIS or invasive cancer, additional resection was performed. We defined negative margins as “no ink on the tumor”^12^ and defined positive margins as DCIS or invasive cancer on the ink surface. Finally, if the resection margin was positive, we performed a re-operation.

### Statistical analysis

The primary outcome of the study was to identify the localization rate using ICG-F in those with early breast cancer undergoing BCS. The secondary outcomes were to compare the safe resection margins for patients with breast cancer who received ICG-F marking versus sono-guided skin marking in BCS and to analyze the safety of the ICG-F marking procedure. We accessed the status of frozen resection margins, the type of intraoperative positive margin, additional resection needed during the operation, final pathologic margin status and re-operation rate. All patients were followed up to evaluate for complications such as skin color changes and necrosis immediately after surgery, from day 7 to day 21, and then again from day 3 to the 6-month postoperative period.

**Figure 3:**
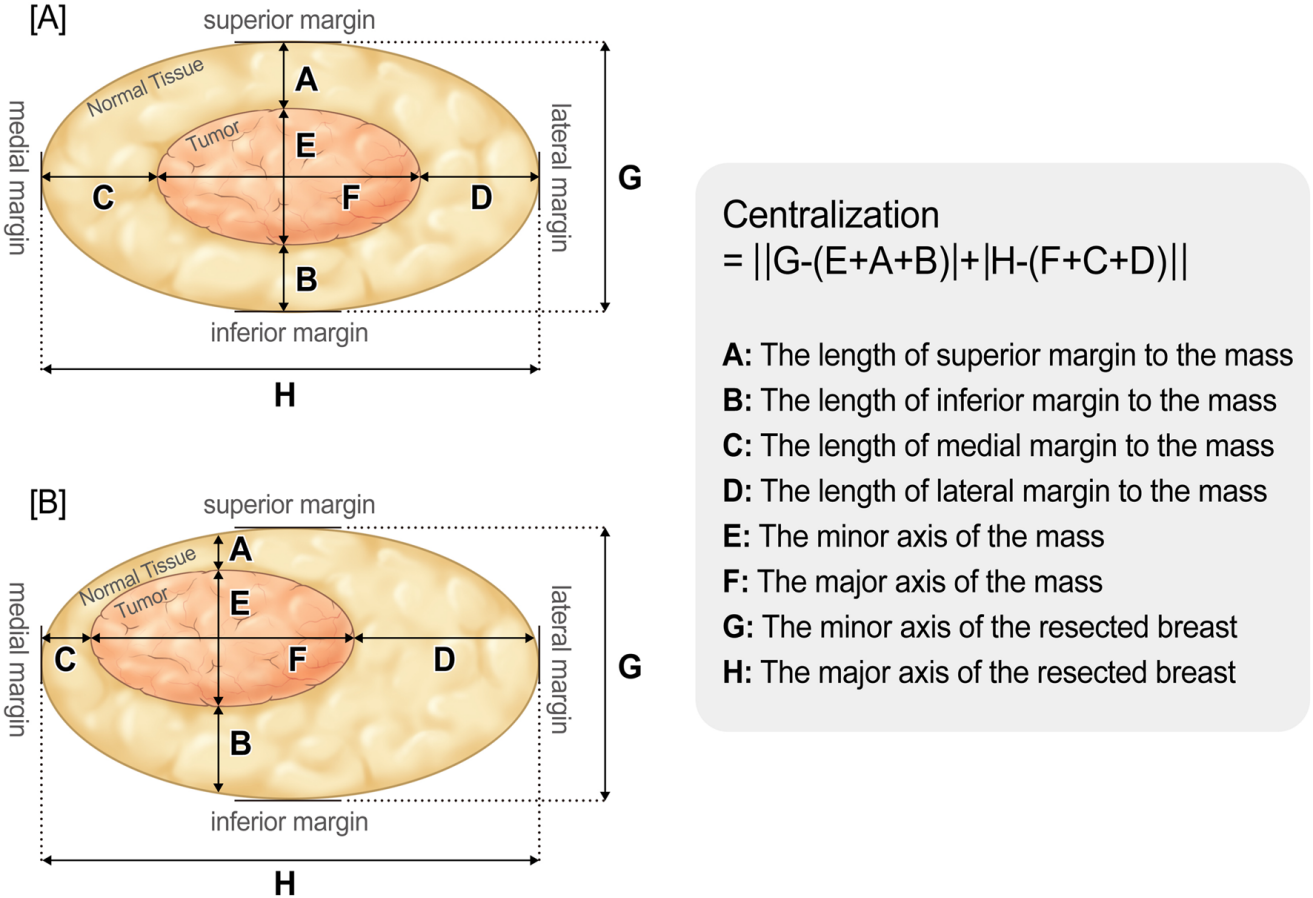
The lesion centralization (diagram). Diagram illustrating the current model for the lesion centralization. The formula of the centralization is next. *Centralization* = *||the major axis of the resected breast* *–* (*the major axis of the mass* + *the length of superior margin to the mass* + *the length of inferior margin to the mass*)*|* + *|the minor axis of the resected breast-*(*the minor axis of the mass* + *the length of medial margin to the mass* + *the length of lateral margin to the mass*)*||.* The closer the calculated value was to zero, the more the lesion was centralized. “A” model was more centralized than “B” model. This work was drawn by the Creative Media Service in National Cancer Center Korea.

The baseline characteristics were performed using the Chi-square test, Fisher’s exact test, and two sample *t* test. Comparison of the distribution between the ICG-F and sono-guided skin marking was performed using the Chi-square test, Fisher’s exact test, and Wilcoxon rank-sum test. We defined lesion centralization as follows (also shown in Fig. 3). The closer the value was to zero, the more the lesion was centralized.

*Centralization* = *||the major axis of the resected breast* *–* (*the major axis of the mass* + *the length of superior margin to the mass* + *the length of inferior margin to the mass*) *|* + *|the minor axis of the resected breast* *−*  (*the minor axis of the mass* + *the length of medial margin to the mass* + *the length of lateral margin to the mass*)*||.*

Statistical significance was set at *p* < 0.05 for all analyses. All statistical analyses were performed using SAS, version 9.4 (SAS Institute Inc., Cary, NC, USA).

### Ethical approval

This study was approved by the Institutional Review Board at the National Cancer Center of Korea (No. NCC2016-0071). All participants singed the informed consent prior to enrolling in this study. All experimental protocols were approved by the same Institutional Review Board and all methods were carried out in accordance with relevant guidelines and regulations.

## Results

### Characteristics of patients

The study enrolled 414 patients. Of them, 114 were in the ICG-F marking group and 300 were in the sono-guided skin marking group. Table 1 shows the clinical characteristics of the patients. The mean ages of the two groups were 52.43 ± 9.75 in the ICG-F marking group and 54.73 ± 10.13 in sono-guided skin marking group (*p* = 0.04). T1 was the most common pathological stage in both groups (74.6% in the ICG-F marking group vs 58.0% in the sono-guided skin marking group, *p* < 0.01). N0 was the most common pathological stage in both groups (84.2% in the ICG-F marking group vs. 75.7% in the sono-guided skin marking group *p* = 0.01).

**Table 1:** Baseline characteristics of the study population.

Characteristics	Total	ICG-F + SONO	SONO	*p* value
	(*n* = 414) *n* (%)	(*n* = 114) *n* (%)	(*n* = 300) *n* (%)	
Mean age (years)	54.1 ± 10.07	52.43 ± 9.75	54.73 ± 10.13	0.04
Tumor size (cm)	1.5 (0.1–7.5)	1.3 (0.1–5.0)	1.6 (0.1–7.5)	< 0.01
**Histologic type**				
DCIS	50 (12.1)	12 (10.5)	38 (12.7)	0.55
Invasive	364 (87.9)	102 (89.5)	262 (87.3)	
**Axillary surgery**				
NA	15 (3.6)	7 (6.1)	8 (2.7)	0.14
SLNB/ALNS	399 (96.4)	107 (93.9)	292 (97.3)	
**Pathologic T stage**				
Tis	50 (12.1)	12 (10.5)	38 (12.7)	< 0.01
T1	259 (62.6)	85 (74.6)	174 (58.0)	
T2	105 (25.4)	17 (14.9)	88 (29.3)	
**Pathologic N stage**				
Nx	15 (3.6)	7 (6.1)	8 (2.7)	0.01
N0	323 (78)	96 (84.2)	227 (75.7)	
N1	66 (15.9)	11 (9.7)	55 (18.3)	
N2	9 (2.2)	0 (0.0)	9 (3.0)	
N3	1 (0.2)	0 (0.0)	1 (0.3)	
**ER**				
Negative	81 (19.6)	16 (14.0)	65 (21.7)	0.08
Positive	333 (80.4)	98 (86.0)	235 (78.3)	
**PR**				
Negative	138 (33.3)	34 (29.8)	104 (34.7)	0.35
Positive	276 (66.7)	80 (70.2)	196 (65.3)	
**HER 2**				
Negative	137 (33.1)	96 (84.2)	101 (33.7)	0.51
Equivocal	143 (34.5)	16 (14.0)	99 (33.0)	
Positive	72 (17.4)	0 (0.0)	51 (17.0)	
NA	62 (15.0)	2 (1.8)	49 (16.3)	

*ICG-F* indocyanine green fluorescence, *SONO* sonography, *DCIS* ductal carcinoma in situ, *NA* not access, *SLNB* sentinel lymph node biopsy, *ALNS* axillary lymph node sampling, *T* tumor, *N* nodal, *ER* estrogen receptor, *PR* progesterone receptor, *HER2* human epidermal growth factor receptor 2.

Histologic type, axillary surgery, hormone receptor status, and human epidermal growth factor receptor 2(HER 2) status did not differ significantly between the two groups. The mean tumor size was 1.3 cm in the ICG-F marking group and 1.6 cm in the sono-guided skin marking group (*p* < 0.01, Table 1).

### Localization by US-guided ICG-F marking

The rate of localization was 100% in both groups: the ICG-F marking group (114/114) and the sono-guided skin marking group (300/300) (Table 2). The mean approach time for resection of the lesion ICG -F using group was about 13 (4–28) min. During the follow-up evaluations after the operation using ICG-F, there were no complications such as skin staining or skin necrosis or allergic reactions related to the procedure in all patients.

**Table 2:** Surgery results for both groups.

Characteristics	Total	ICG-F + SONO	SONO	*p* value
	(*n* = 414)	(*n* = 114)	(*n* = 300)	
	*n* (%)	*n* (%)	*n* (%)	
Localization rate	414 (100)	114 (100)	300 (100)	1
**Frozen resection margin**				
Negative	327 (79.0)	102 (89.5)	225 (75.0)	< 0.01
Positive	87 (21.0)	12 (10.5)	75 (25.0)	
**Intra-op margin ( +) type**				
Precancerous lesion / LCIS	52 (59.8)	9 (75.0)	43 (57.3)	0.60
DCIS	23 (26.4)	2 (16.7)	21 (28.0)	
Invasive	12 (13.8)	1 (8.3)	11 (14.7)	
**Intra-op further resection**				
No	334 (80.7)	104 (91.2)	230 (76.7)	< 0.01
Yes	80 (19.3)	10 (8.8)	70 (23.3)	
**Permanent resection margin**				
Negative	366 (88.4)	110 (96.5)	256 (85.3)	< 0.01
Positive	48 (11.6)	4 (3.5)	44 (14.7)	
**Permanent margin positive type**				
Precancerous lesion/LCIS	28 (58.3)	1 (25.0)	27 (61.4)	0.11
DCIS	12 (25.0)	1 (25.0)	11 (25.0)	
Invasive	8 (16.7)	2 (50.0)	6 (13.6)	
**Re-operation**				
No	397 (95.9)	109 (95.6)	288 (96.0)	0.79
Yes	17 (4.1)	5 (4.4)	12 (4.0)	
**Re-operation pathology**				
No residual	8 (47.1)	4 (80.0)	4 (33.3)	0.13
Residual	9 (52.9)	1 (20.0)	8 (66.7)	
**Resection size (gross) (cm**^3^**)**				
Total	97.47 (12.22–722.50)	75.11 (12.22–325.12)	105.15 (17.61–722.50)	< 0.01
**T stage**				
Tis	92.25 (12.22–722.5)	60.04 (12.22–189.00)	95.70 (18.00–722.50)	0.05
T1	84.5 (13.16–480.00)	69.00 (13.16–325.12)	97.95 (17.61–480.00)	< 0.01
T2	140.25 (22.68–432.00)	117.00 (54.00–252.00)	152.00 (22.68–432.00)	< 0.01
**Resection mass size (gross) (cm**^3^**)**				
Missing cases	51	14	37	
Total	2.16 (0.01–84.00)	1.20 (0.01–28.80)	2.60 (0.04–84.00)	< 0.01
**T stage**				
Tis	1.47 (0.02–84.00)	0.50 (0.02–7.50)	1.65 (0.25–84.00)	< 0.01
T1	1.49 (0.01–60.78)	1.08 (0.01–28.80)	1.67 (0.04–60.78)	0.10
T2	7.91 (0.36–47.43)	6.72 (3.60–22.04)	8.10 (0.36–47.43)	0.12
**Centralization**				
Missing cases	34	6	28	
	3.35 (0.30–27.70)	2.40 (0.30–10.40)	3.70 (0.30–27.70)	< 0.01

*ICG-F* indocyanine green fluorescence, *SONO* sonography, *LCIS* lobular carcinoma in situ, *DCIS* ductal carcinoma in situ.

### Comparison of the safe resection margin

Table 2 shows the surgery results including resection margin status, type of positive resection margin, and re-operation status.

The positive rate of frozen resection margin was 10.5% using ICG-F and 25% using sono-guided skin marking. In the ICG-F marking group, the positive rate of frozen resection margin was statistically significant low (*p* < 0.01). The rate of intraoperative additional resection was significantly lower in the ICG-F marking group than that in the sono-guided skin marking group (8.8% vs. 23.3%, *p* < 0.01). When comparing the type of positive margins, both groups had more DCIS than invasive cancer. There was no statistically significant difference. The rates of re-operation were similar between the two groups (4.4% in the ICG-F marking group and 4.0% in sono-guided skin marking group, *p* = 0.79%).

### Centralization of cancer lesion

Table 2 showed that the removed breast volume was 75.11 cm^3^ in the ICG-F marking group and 105.15 cm^3^ in sono-guided skin marking group. Those in the sono-guided skin marking group had more volume removed than that in the ICG-F marking group, and the difference was statistically significant. When the gross size of breast cancer was compared between groups, there was a significantly larger average mass size (2.6 cm^3^) in the ICG-F group compared to that in the sono-guided skin marking group (1.2 cm^3^). The lesion centralization was found to be higher in ICG-F marking group than that in the sono-guided skin marking group, and the difference was statistically significant (2.40 in the ICG-F marking group vs 3.70 in sono-guided skin marking group, *p* < 0.01, Table 2).

## Discussion

In the current study, we investigated whether ICG-F was accurate for performing BCS. The results showed that both ICG-F marking and sono-guided skin marking groups confirmed 100% localization. The centralization of the lesion was better when ICG-F marking was used compared to sono-guided skin marking.

ICG, which acts on the blood lipoproteins, is a nontoxic and nonionizing agent with a short half-life in the blood circulation. Usability is increasing in various clinical fields such as cancer treatment and various types of surgery^13^. Oncologic studies using ICG-F to harvest sentinel lymph nodes have been reported^11,14,15^. The feasibility of sentinel lymph node biopsy using ICG-F for patients with early and advanced breast cancer after neoadjuvant therapy has been demonstrated^11,16^.

In the case of localization using ultrasonography, one of the traditional methods, it is difficult to distinguish microcalcification lesions, to discriminate the depth difference from the skin incision to the main lesion in patients with large breast volume, and to reconfirm using ultrasonography during surgery due to the air layer after skin incision^17^. Wire localization, one of the other traditional methods, has technical limitations as wires often get displaced and patients experience discomfort after localization.

In the National Comprehensive Cancer Network guidelines for performing BCS for invasive cancer, the “no ink on tumor” method is accepted as a pathological measure of negative margins. If the tumor is positive on the resection surface, the local–regional recurrence rate increases. Positive margins demand re-excision or additional radiotherapy to the tumor bed. In a previous case report on non-palpable breast lesion localization, ICG-F guided occult lesion localization (IFOLL) demonstrated successful surgical excision indicating that IFOLL was a clinically acceptable procedure^8^. A randomized clinical trial using ICG-F localization for non-palpable lesions showed that the rate of clear margins was significantly high (87.5% in the ICG-F group vs. 63.3% in the wire localization group, *p* = 0.026)^10^. Another study on the excision of non-palpable breast cancer using ICG-F confirmed localization of 100% and a safe margin at about 95%^9^. Previously studies have examined injections into the center of the tumor using ICG-F, but we approached the peritumor area using ICG-F injections, and confirmed that the localization could be successful with such an approach. This method has the advantage of being able to inject into the surgical range planned by the operator. The negative margin rate was significantly higher than that of skin marking using ultrasonography (95.6% in the ICG-F marking group vs. 85.3% in sono-guided skin marking group, *p* = < 0.01).

BCS is considered a minimally invasive surgery; however, the results of cosmetic satisfaction with the surgery vary. In previous studies, cosmetic satisfaction of patients who underwent BCS was about 80%. Having a large amount of breast tissue resected is a major determinant of lowering cosmetic satisfaction outcomes^18,19^. Surgeons are concerned about how to remove normal breast tissue to a minimum, taking the cosmetic aspect into consideration while completely removing the tumor. A randomized control study reported the superiority of sono-guided BCS comparing traditional palpitation-guided surgery to improve cosmetic outcome due to smaller resection volume and improvement of margin status^20^. Our study showed that the resection volume was significantly small in ICG-F marking groups than sono-guided skin marking groups Above all, the lesion centralization was found to be significantly higher in the ICG-F group than in the sono-guided skin marking group. The use of ICG-F may be safer for removing the tumor and may help prevent removing normal tissue.

Our study had several limitations. First, the comparison time between the two groups was different. The ICG-F marking group was planned as a prospective study, while the sono-guided skin marking group was compared using retrospective data. However, we think the error that can occur over time may be reduced, because the patients who had surgery were selected by the same doctors. Second, this study was focused objective results using ICG-F, and subjective factors such as patient satisfaction and quality of life were not investigated. Third, there were more advanced cases in the sono-guided skin marking group compared to the ICG-F marking group. The larger the size of the tumor, the more volume in the resected breast. We compared T stages and confirmed that the resected volumes in the ICG-F marking group were significantly smaller. Fourth, the criteria for negative margins of the lesion has been changed to “no ink tumor” since 2014. In our study, the control group included patients before 2014; the patients before 2014 were also evaluated for margin status by the changed criteria since 2014. To overcome this limitation, we checked the centralization of the lesion. Fifth, institutions that use this method may be limited since equipment using ICG-F is not yet popularized. However, this study shows that it is one of the various approaches to localization and it seems that it can be applied in institution which plan to set these equipment up in the future. Finally, this study was a single-center study. In the future we need to consider a multicenter, randomized trial to further evaluate the ICG-F marking method.

## Conclusion

This study demonstrated that BCS using ICG-F was comparable with that of the sono guided-skin marking method. We verified that ICG-F injected marking around the tumor is a non-inferior approach compared to sono guided-skin marking. The ICG-F method could be an easy approach to take for marking in a clinical setting and may provide more accurate and safe resection of the tumor in BCS.
